# *De Novo* Gene Emergence: Summary, Classification, and Challenges of Current Methods

**DOI:** 10.1093/gbe/evaf197

**Published:** 2025-10-23

**Authors:** Anna Grandchamp, Margaux Aubel, Lars A Eicholt, Paul Roginski, Victor Luria, Amir Karger, Elias Dohmen

**Affiliations:** Aix Marseille University, INSERM, TAGC institute, UMR_S1090, 13288 Marseille, France; Institute for Evolution and Biodiversity, University of Münster, Münster 48149, Germany; Institute for Evolution and Biodiversity, University of Münster, Münster 48149, Germany; Institute for Integrative Biology of the Cell (I2BC), Université Paris-Saclay, CEA, CNRS, Gif-sur-Yvette 91198, France; Department of Neuroscience, Yale School of Medicine, New Haven, CT 06510, USA; Department of Systems Biology, Harvard Medical School, Boston, MA 02115, USA; Division of Genetics and Genomics, Boston Children’s Hospital, Harvard Medical School, Boston, MA 02115, USA; IT-Research Computing, Harvard Medical School, Boston, MA 02115, USA; Institute for Evolution and Biodiversity, University of Münster, Münster 48149, Germany

**Keywords:** de novo gene emergence, annotation format, comparative genomics, comparative transcriptomics

## Abstract

A novel mechanism of *de novo* gene origination from nongenic sequences was first proposed in the early 2000s. Subsequent studies have since provided evidence of *de novo* gene emergence across all domains of life, revealing its occurrence to be more frequent than initially anticipated. While studies mainly agree on the general concept of *de novo* emergence from nongenic DNA, the exact methods and definitions for detecting *de novo* genes differ significantly. Here, we provide a comprehensive step-by-step description of the most commonly used methods for *de novo* gene detection. In addition, we address the limitations of nomenclature and detection methods and clarify some complex concepts that are sometimes misused. This review is accompanied by the publication of a *de novo* gene annotation format to standardize the reporting of methodology, enable reproducibility and improve the comparability of datasets.

Significance While it is now widely accepted that new genes can emerge from previously noncoding DNA, researchers still lack consistent methods and definitions for identifying these “*de novo*” genes. This review lays out the most common techniques for detecting *de novo* genes, highlights their differences and limitations, and introduces a standard annotation format to make future studies more comparable. By establishing clearer methodological guidelines, this work helps unify a rapidly growing field and improves our ability to study how entirely new genes arise.

## Introduction

**Fig. 1.**
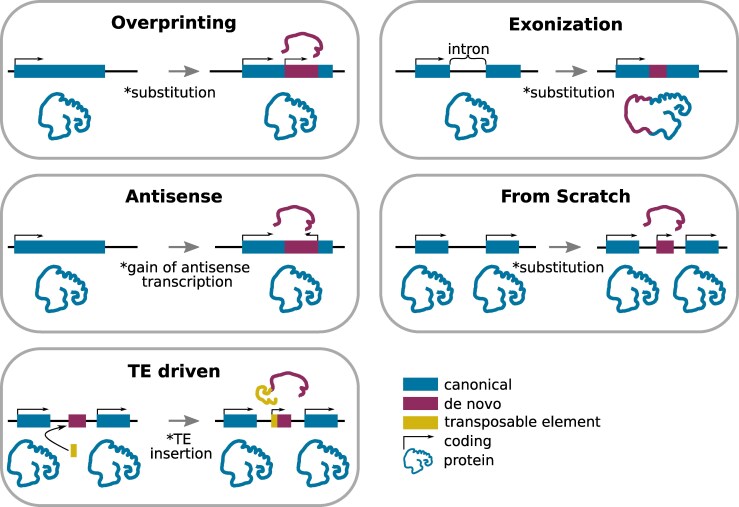
Main mechanisms of genes emergence. The figure represents a simplified representation of the concepts discussed.

Throughout evolution, genes can arise by “recycling the old”, emerging from pre-existing genetic material through mechanisms (Fig. 1) such as duplication (Ohno 2013), exon shuffling (Gilbert 1978), horizontal gene transfer (Freeman 1951), retrotransposition (Baltimore 1970; Temin et al. 1970; Coffin and Fan 2016) and gene fusion (Nowell and Hungerford 1960; Mitelman et al. 2007).

However, it is now well documented that new genes can also emerge *de novo*, through a series of mutations in the noncoding genome (Begun et al. 2006, 2007; Heinen et al. 2009; Rancurel et al. 2009; Toll-Riera et al. 2009; Tautz and Domazet-Lošo 2011; Neme and Tautz 2013; Zhao et al. 2014; Xia et al. 2025). Several mechanisms of *de novo* gene emergence have been identified (Fig. 1):

- **Overprinting.** Overprinting refers to the emergence of an alternative ORF in a +1 or +2 frame in forward direction of an existing gene, leading to the translation of a protein entirely different from the canonical protein (Keese and Gibbs 1992; Rogozin et al. 2002; Pavesi 2006; Delaye et al. 2008; Carter et al. 2013). It can also emerge upstream the ancestral open reading frame (ORF), for example in the 5’ UTR (Renz et al. 2020), or downstream the ancestral ORF, in the 3’UTR (Wu et al. 2020).
- **Exonization.** Exonization characterises mutations inside a gene, leading to the gain of a new exon, and potentially to a new ORF (Makałowski et al. 1994; Sorek 2007; Cai et al. 2008; Schmitz and Brosius 2011). It can occur for example by the the loss of a splicing sites, converting an original intron into an exon (Roy 2004; Moldón and Query 2010; Koralewski and Krutovsky 2011).
- **Antisense.**  *De novo* genes can also emerge overlapping existing genes, but on the opposite strand (Merino et al. 1994; Barman et al. 2019; Iyengar et al. 2024). Transcribed antisense *de novo* emerged ORFs have been reported to generate functional proteins (Ardern et al. 2020; Thomas et al. 2023) or regulate translation efficiency (Liang et al. 2016).
- **From scratch in intergenic region.** In order to emerge in an intergenic region, the future gene needs to acquire everything: a transcription event, an ORF, the ability to be translated, a certain stability in the untranslated regions (UTRs), eventually introns, etc. However, such challenging mechanisms of new gene emergence have been heavily reported (McLysaght and Guerzoni 2015; Schlötterer 2015; Heames et al. 2020; Papadopoulos et al. 2021; Iyengar and Bornberg-Bauer 2023; Lombardo et al. 2023).
- ***De novo* gene birth driven by Transposable Elements (TEs).** Cumulative evidence tend to show that *de novo* gene birth can also be linked to the insertion of a TE inside an intergenic sequence (Cordaux et al. 2006; Jin et al. 2021; Delihas 2022; Lee et al. 2022; Lebherz et al. 2024a; Uz-Zaman et al. 2024).

Despite their different origins, these mechanisms share a common feature: the *de novo* gene or its encoded protein lack detectable similarity to any other known gene or protein (McLysaght and Hurst 2016). However, also other mechanisms like duplication and divergence can generate genes without detectable similarity (Tautz and Domazet-Lošo 2011)

Therefore, one of the major challenges in *de novo* gene research is to accurately determine whether a gene truly emerged *de novo* or has arisen through other mechanisms (Tautz and Domazet-Lošo 2011; Casola 2018). For example, after a duplication event, the duplicated gene copy can evolve rapidly and its sequence can undergo significant rearrangement (Innan and Kondrashov 2010) so that it is misidentified as originating *de novo*. The work of (Casola 2018) shed light on inaccuracies in the validation of *de novo* gene emergence, and was followed by significant advances in the precision of detection and the design of pipelines for confirming *de novo* origins. As methods for *de novo* gene detection and validation have become more sophisticated, proper annotation of the methodology has become essential (Moyers and Zhang 2016, 2017; Weisman et al. 2022).

In the field of *de novo* gene research, the mechanisms and definitions of *de novo* emergence remain a pivotal yet variable factor in identifying such genes. Across studies, authors have incorporated diverse evolutionary stages and criteria (Keeling et al. 2019; Weisman 2022), such as varying thresholds for how much of a gene must have originated *de novo* (McLysaght and Hurst 2016), and differing standards to establish the absence of homology (Casola 2018; Vakirlis et al. 2020; Weisman et al. 2022). Although this conceptual diversity has enriched the field, it has also introduced ambiguities that challenge the consistency and comparability of results (Schmitz et al. 2018; Dohmen et al. 2025).

This diversity arises from several factors. First, the field of de novo gene birth is still relatively new, which has led researchers to explore a variety of methods to investigate different mechanisms and address distinct questions. For example, studying the emergence of de novo transcription requires different approaches than examining the early fixation of genes across multiple taxa. Additionally, the data used vary between studies, depending on the species of interest. For instance, nonmodel organism genomes are less well-characterized and annotated than those of humans (Vakirlis and Kupczok 2024), and they typically lack extensive transcriptomic data. At this stage, maintaining an openness to exploring various methodologies remains crucial, but addressing these semantic and conceptual divergences is equally important to advance the field and improve the integration of findings across studies.

In this review, we outline the key steps that currently allow for accurate discrimination between *de novo* genes and genes arising from other mechanisms. We also highlight the main methodological differences between studies and address the challenges and controversies that remain with current approaches. In this article, we define a *de novo* gene as one that emerges from a previously noncoding region of the genome through mutations. Notably, the detection methods we describe assume the presence of a transcribed ORF, although the definition of a gene does not always require it to be protein-coding (Orgogozo et al. 2016; Li and Liu 2019). Any deviations from this definition in specific cases are explicitly noted in the text.

The main goal of this review is to provide a comprehensive overview of current methodologies, their strengths, and weaknesses to allow for informed decisions about the most suitable approach for a given research question in the field of *de novo* research. It is not meant to recommend one method or tool over another, but rather to be able to identify the best approach for a given context and input data. As a consequence of the differences in methods and approaches identified here, and as a guidance for choices in *de novo* gene identification pipelines, we have developed an annotation format to standardize the reporting of the methodology used, that allows for easy comparison between datasets (Dohmen et al. 2025).

## Tools and Techniques in the Computational Detection of *De Novo* Genes

### Choice of Candidate Genes

The initial step in the identification of *de novo* genes or proto-genes is the selection of candidate genes from a given species, population or individual. Unless a subset of genes has already been identified as candidate *de novo* genes, often, the entire genome or transcriptome is screened to distinguish *de novo* genes from others. Two distinct approaches are commonly employed in the identification of *de novo* genes: the first involves the assessment of annotated genes within an annotated genome, while the second entails the evaluation of ORFs extracted from a transcriptome, sometimes accompanied by the validation of translation.

#### Candidate Genes from Annotated Genomes

**Fig. 2.**
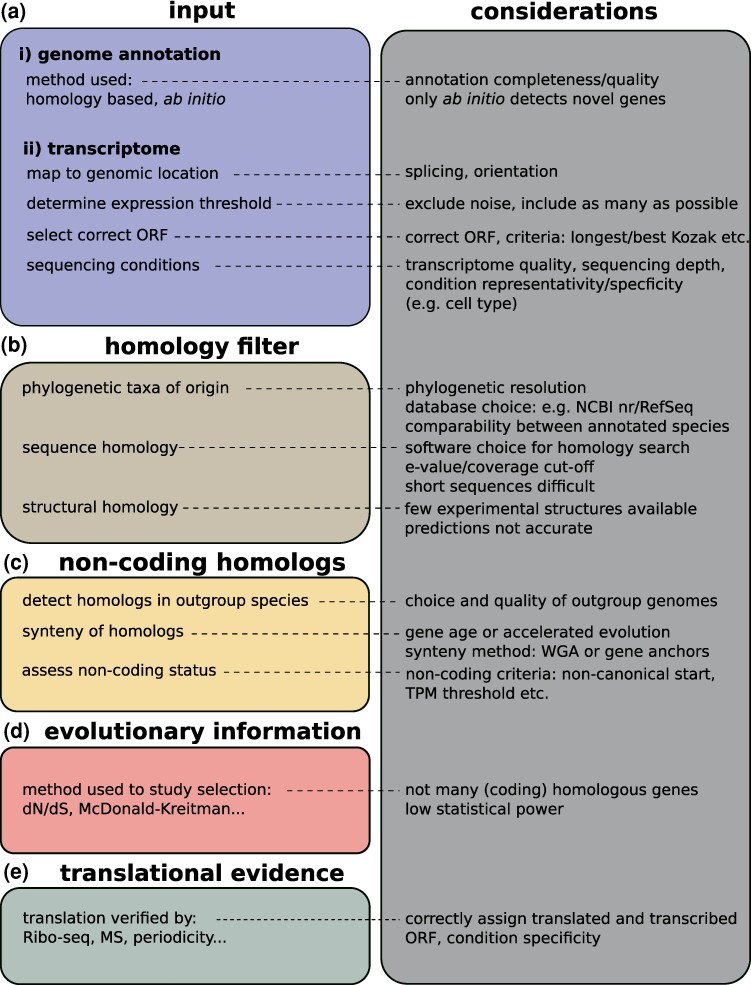
Considerations for general approaches and standards in *de novo* gene research. Related literature can be found in Table 1.

The identification of potential *de novo* genes in an annotated genome consists in determining which annotated genes correspond to genes that have potentially emerged *de novo* in a specified taxonomic group. Therefore, following the annotation, all identified genes will be considered as candidates for a *de novo* origin analysis. Annotated genomes can be obtained from public databases, such as NCBI (Schoch et al. 2020), or they can be obtained through genome assembly from DNA-seq data followed by application of a gene annotation pipeline. In the latter case, it is necessary to annotate the genomes. In the specific context of *de novo* gene detection, a combination of homology-based approaches (Söding 2005; Eddy 2009) with *ab initio* approaches (Wang et al. 2004; Scalzitti et al. 2020; Baker et al. 2023) is encouraged, given that the latter relies on algorithms that recognize various genic properties within a genome even without gene homology (Fig. 2a, Table 1).

**Table 1.** Considerations and related literature for general approaches and standards in *de novo* gene research.

section	considerations and literature
genome annotation method	**completeness/quality** (Casola 2018; Vakirlis and McLysaght 2018; Weisman et al. 2022), ***ab initio* for novel genes** (Wang et al. 2004; Scalzitti et al. 2020; Baker et al. 2023)
map transcriptome to genome	**splicing, orientation** (Iyengar et al. 2024)
determine expression threshold	**exclude noise but not low expression, consider different thresholds** (Heames et al. 2020; Blevins et al. 2021; Grandchamp et al. 2023a; Lombardo et al. 2023)
select correct ORF	**criteria: length/Kozak/**… (Xu et al. 2010; Schmitz et al. 2018; Dowling et al. 2020; Heames et al. 2020; Whiffin et al. 2020; Blevins et al. 2021; Iyengar and Bornberg-Bauer 2023)
sequencing conditions	**transcriptome quality, sequencing depth, condition specificity (e.g. cell type)** (Toll-Riera et al. 2009; Schlötterer 2015; Blevins et al. 2021)
phylogenetic taxa of origin	**phylogenetic resolution** (Li et al. 2021), **database choice** (Moyers and Zhang 2015; Vakirlis and McLysaght 2018; Weisman et al. 2020)
sequence homology	**software choice** (Altschul et al. 1990; Finn et al. 2011; Buchfink et al. 2021), **sequence similarity cutoff, especially short sequences difficult** (Domazet-Loso and Tautz 2003; Moyers and Zhang 2015, 2016, 2017, 2018; Vakirlis et al. 2020; Weisman et al. 2020)
structural homology	**few experimental structures, predictions not accurate** (Aubel et al. 2023; Middendorf and Eicholt 2024)
detect homologs in target species	**choice/quality of target genomes** (Moyers and Zhang 2015; Vakirlis and McLysaght 2018; Weisman et al. 2020)
synteny of homologs	**gene age or accelerated evolution** (Ranz et al. 2001; Zdobnov et al. 2002; Casola 2018; Vakirlis et al. 2020; Weisman et al. 2020), **synteny method** (Casola 2018; Vakirlis et al. 2020; Roginski et al. 2024) **WGA** (Peng and Zhao 2024), **phylostratigraphy** (Ranz et al. 2001; Zdobnov et al. 2002; Moyers and Zhang 2015, 2016, 2017, 2018; Prabh and Rödelsperger 2019)
assess noncoding status	**criteria: noncanonical start, TPM threshold etc.** (Roginski et al. 2024; Vakirlis et al. 2024)
method used to study selection	**limited number of (coding) homologs** (Zhao et al. 2014; Chen et al. 2015; Schlötterer 2015; Gubala et al. 2017; Rivard et al. 2021; Broeils et al. 2023)
translation verified	**correctly assign ORF, condition specificity** (Wilson and Masel 2011; Ruiz-Orera et al. 2014; Vakirlis et al. 2018; Ruiz-Orera and Albà 2019; Zhang et al. 2019; Patraquim et al. 2020; Papadopoulos et al. 2021; Patraquim et al. 2022; Papadopoulos et al. 2024)

#### Candidate Genes from Transcriptomes

Another option for the detection of candidate *de novo* genes is to analyze transcripts from one or multiple transcriptomes. Starting from a transcriptome implies access to an annotated genome. However, in this case, annotated genes are regarded as canonical genes rather than putative de novo genes. Instead, the transcriptome is screened for transcribed ORFs that do not overlap with annotated genes. These unannotated transcribed ORFs are then considered as putative *de novo* genes. The scenario in which no reference genome is available is also discussed below, but it requires particular caution. This approach involves more initial steps described below, but it likely allows for the detection of *de novo* genes in their early stages of emergence, such as proto-genes (Carvunis et al. 2012) or *de novo* ORFs (Grandchamp et al. 2023b). The steps described in the following assume that the transcriptome has already been assembled based on a reference genome, using reference-based algorithms (Kovaka et al. 2019; Raghavan et al. 2022). If a transcriptome has been assembled *de novo*, the primary deviation from the described method resides in the identification of genomic locations of the ORFs. If no reference genome exists for the query species, the genomic location of ORFs may lack precision, which may lead to misleading interpretations in subsequent steps of the pipeline. We encourage authors pursuing such an approach to draw conclusions carefully. Finally, another possibility would be to search for ORFs in a *de novo* assembled transcriptome and directly look for homologous sequences in target databases, without relying on genomic information. To our knowledge, no study has implemented such a pipeline, although it may be the only feasible option when genome data is extremely limited. However, using the term *de novo* genes to describe genes identified through such a pipeline is misleading. Instead, we suggest using more cautious terminology—such as specifying their putative status—and clearly stating the limitations of the approach.

##### Selection of transcripts based on genomic location

*De novo* genes can be located in various genomic regions, including intergenic spaces, introns, overlapping existing genes in a different frame or antisense orientation, within UTRs, or other nongenic locations. Depending on the investigated *de novo* emergence mechanism(s), certain transcripts (or ORFs) may be excluded from the analysis. Utilizing tools such as BEDtools (Quinlan and Hall 2010) facilitates the determination of the genomic overlap of the transcripts, and the choice of which transcripts will be retained as candidates for further analyses. This step can also be conducted using ORFs instead of transcripts, after ORF detection in transcripts. The appropriate use of BEDTools depends on the type of RNA-seq library. If the library is strand-specific, BEDTools should be used with strand-aware operations. In contrast, strand-awareness is not applicable for unstranded libraries. If the RNA-seq data is not stranded, it is not possible to determine the strand on which an ORF is located, which can significantly affect the conclusions. For example, an ORF overlapping an existing gene would be considered antisense if it originates from the opposite strand, whereas it would be classified as an overprinted gene if it arises on the same strand.

##### Detection of ORFs in a transcriptome

After filtering transcripts based on their genomic location, the selected spliced transcripts are scanned for ORFs. Various software tools are available for extracting ORFs from a transcriptome, with one notable example being EMBOSS getorf (Rice et al. 2000). This tool conveniently provides information on the position of the ORF in the spliced transcript and its direction (forward or reverse). However, ORFs that extend to the end of a transcript without ending with a stop codon are also retrieved, which might be considered as erroneous and should be removed.

In order to extract the ORFs relevant to a given biological question, a number of steps must be followed:

1. If the RNA is stranded, detected antisense ORFs may be erroneous and should be regarded with caution.
2. Multiple transcripts may correspond to the spliced product of a single gene, and some might overlap (Lebherz et al. 2024b). In such cases, removing duplicated ORFs shared among transcripts spliced from the same genomic location may be necessary.
3. The majority of transcripts contains multiple ORFs, and the choice of the ORF(s) within a transcript depends on the biological question, and various choices are valid (Xu et al. 2010).

##### Choice of Coding ORFs

When starting from a transcriptome with transcripts containing several ORFs, the selection of which ORFs to keep for further steps is decided by the investigator. Until recently, ORFs were typically considered potentially coding only if their size exceeded 300 nucleotides, a criterion implemented in algorithms such as those used by the Functional ANnoTation Of the Mammalian Genome (FANTOM) (Dinger et al. 2008; Leong et al. 2022). However, micropeptide and short *de novo* genes are known to have coding potential (Patraquim et al. 2022; Vakirlis et al. 2022; Sandmann et al. 2023), and *de novo* genes have been shown to be shorter (Guo et al. 2007; Toll-Riera et al. 2009; Palmieri et al. 2014). Various software tools have been developed to determine which ORF should be considered as the coding one in canonical genes, using approaches primarily based on protein homology (Vitting-Seerup et al. 2014; Kang et al. 2017; Varabyou et al. 2023). Nevertheless, even for canonical genes, the definition and number of coding ORFs are under revision, as the coding potential of genes has been shown to be significantly underestimated (Wright et al. 2022; Ardern 2023).

In transcripts, all ORFs within a size limit can be considered. The majority of studies opt for the longest ORF (Xu et al. 2010; Dowling et al. 2020), which is also the default option for annotating protein-coding regions in most software (Rombel et al. 2002; Wang et al. 2013). Some studies only consider the first upstream ORF (uORFs) (Whiffin et al. 2020). Other studies consider the ORFs with the highest Kozak score (Kozak 1989; Xu et al. 2010), indicating the highest likelihood of translation, or ORFs including surrounding untranslated regions (UTRs), since UTRs play crucial roles in translation initiation and transcript stability (Chatterjee and Pal 2009; Matoulkova et al. 2012).

Importantly, the detection of ORFs with coding potential does not guarantee a translation event. Several studies have reported only a weak correlation between transcript expression levels and protein abundance (Gry et al. 2009; Koussounadis et al. 2015; Liu et al. 2016). This emphasizes that a transcribed ORF is strongly dependent on posttranscriptional and translational regulatory mechanisms for translation, which is difficult to predict without experimental evidence.

##### Selection of an expression threshold

Most studies include only the ORFs from transcripts that reach a minimum level of expression, which is typically determined by the transcripts per million (TPM) threshold. A threshold of 0.5 TPM has been adopted by numerous studies (Petryszak et al. 2016; Poretti et al. 2023; Vara et al. 2024) as specified by EMBL (Stoesser et al. 2002) as the minimal expression threshold. When assembling transcriptomes, low-expressed transcripts are often removed from the process as they are suspected to represent background noise (Janssen et al. 2023). However, emergence of low-expressed transcripts could be a step towards *de novo* gene emergence, and such transcripts might be important to study. The hypothesis that transcripts are produced throughout the entire genome of a species is referred to as pervasive transcription (Clark et al. 2011; Hangauer et al. 2013; Kellis et al. 2014). In cases involving splicing, it is crucial to be cautious when employing a TPM threshold. It is plausible for a gene to express multiple transcripts, where one transcript meets the specified threshold while the others do not.

##### Detection of genomic positions of unspliced transcripts and ORFs

In order to account for splicing events and the subsequent methodological steps, the genomic position of the selected ORFs must be detected. The software BLAT (Kent 2002) is splicing-aware and can be used to map ORFs from a transcriptome to the corresponding genome. However, BLAT has difficulties dealing with short sequences, as *de novo* ORFs often are. Instead of aligning intact ORFs, BLAT overpredicts splicing events by splitting up ORFs to align them to multiple locations in the genome. Other splice-aware software, such as Exonerate (Slater and Birney 2005), can also be used, although they share similar limitations. The most precise method for retrieving the genomic location of an ORF is to extract the coordinates from the transcript it originates from. This accurate approach is only feasible if the transcriptome is assembled using reference-based algorithms. While the genomic coordinates of transcripts are provided by the assembly software, the precise genomic positions of the ORFs within them must be calculated based on the transcript’s genomic location, its exon-intron structure and genomic strand orientation (forward or reverse). To our knowledge, such a step cannot be fulfilled by existing software and requires custom scripts.

After all these steps, all filtered ORFs and/or transcripts can be considered as candidate *de novo* genes and will be used for the next filtering steps.

#### Validation of Translation

To assess whether the selected candidate genes are coding genes, one option is to use experimental validation (Fig. 2e, Table 1). Experimental validation of a gene’s coding status can be performed at the very end of the methodology, when only a subset of genes has been validated as *de novo* genes. However, when starting from a transcriptome, validating translation can be the very first step of the method. In such cases, all translated ORFs detected experimentally are mapped to the corresponding transcriptome (Wacholder et al. 2023) and subsequently sorted through several steps similar to those used in transcriptome analysis (Turcan et al. 2024).

To confirm the coding status of putative *de novo* genes, several new laboratory techniques have proven to be highly effective, particularly for small proteins. Ribosome profiling-based approaches (Ribo-Seq) (Ingolia et al. 2009; Kondo et al. 2010; Ingolia et al. 2011; Bazzini et al. 2014; Chen et al. 2020; Duffy et al. 2022) and mass spectrometry-based approaches (Slavoff et al. 2013; Pauli et al. 2014; Ji et al. 2015) assess the binding of ribosomes to transcribed ORFs or the presence of translated proteins. These two approaches can also be combined for better accuracy (Schlesinger and Elsässer 2022; Wacholder and Carvunis 2023; Andjus et al. 2024).

The search for population genetic signatures can also provide evidence of coding potential, particularly if the ORF is under selection. Tests such as the McDonald-Kreitman test (McDonald and Kreitman 1991), which assess the ratio of nonsynonymous to synonymous polymorphisms (pN/pS), can help determine whether an ORF is subject to selective pressure. However, these tests are often challenging to apply in the context of de novo gene emergence, as they require that the sequences across the population—and ideally in an outgroup species—are coding. This condition is rarely met during the early stages of de novo gene evolution.

#### Genomes or Transcriptomes?

The choice between candidate *de novo* genes from annotated genomes or transcriptomes depends on the biological question being investigated. Candidate genes from an annotated genome provide a high level of confidence about the genic status of the identified *de novo* genes at the end of the pipeline. Evolutionary fixation in a species is more likely for these genes, as their genic structures are apparently stable enough to be recognized by annotation methods. Nevertheless, *de novo* genes that are lacking gene homology or genic structures, such as introns or specific transcription motifs, may not be detected by annotation tools.

Selecting candidate genes from a transcriptome generally results in the identification of a considerably higher number of *de novo* genes compared to candidate genes from an annotated genome. For example, in Roginski et al. (2024), the authors detected 89 *de novo* genes in humans when starting from a genome, while Dowling et al. (2020) identified 2,749 human-specific *de novo* expressed ORFs when starting from a transcriptome. Similarly, Roginski et al. (2024) detected 92 *de novo* genes in *Drosophila melanogaster* by analyzing an annotated genome, while Zheng and Zhao (2022) identified 993 *de novo* genes in the same species using Ribo-seq data mapped to a transcriptome. However, depending on the specific transcriptome and the applied criteria, it is possible that the majority of the detected translated ORFs may not be fixed in the species (Roginski et al. 2024). An automated pipeline to detect very early stages of potential *de novo* genes based on transcriptomic data verifies this higher number of candidates (Grandchamp et al. 2025) when compared with annotated genome-based approaches.

The genic status of *de novo* candidates can be confirmed through the validation of translation as described above and subsequently only considering the translated ORFs. When starting from a transcriptome, one important issue can come from the fact that transcript expression is complicated to characterise, as expression can depend on conditions, tissues, sex, life stage, individuals or populations, among others (Oliva et al. 2020; Nieuwenhuis et al. 2021; Xu et al. 2023; Schneider et al. 2024). Consequently, particular *de novo* genes can be specific to certain conditions or tissues (Fig. 2a, Table 1). The detection of such genes can be more challenging, particularly when their expression levels are low.

### Taxonomic Group of Emergence

A *de novo* gene or expressed ORF may be specific to an individual, a population, a species, or a broader taxonomic group. When starting from a transcriptome, it may also be expressed only under specific conditions, such as in a specific tissue, age or sex. The taxonomic level of emergence can but does not have to be specified in advance, ensuring that only *de novo* genes meeting a particular condition are retained. If a gene is not specific to a single species, population, or condition, but instead is shared among several closely related species, it is referred to as a taxonomically restricted gene. The distinction between *de novo* genes and other genes becomes more challenging when they are shared by several rather than one single species, particularly if they have an evolutionary origin predating a loss of synteny within the taxa to which they belong, and if they exhibit a high mutation rate, although this is likely not frequent (Domazet-Lošo et al. 2017). The more distantly related the species in the taxonomic group are, the more information is lost about *de novo* gene emergence or their mechanism of emergence in general. *De novo* gene birth is easier to identify in taxonomic groups including species that diverged recently, provided that the considered evolutionary time is sufficient to characterize the genicity of the sequences. A large number of studies focuses on species-specific *de novo* genes (Zhao et al. 2014; Schmitz et al. 2018; Zhang et al. 2019; Broeils et al. 2023; Grandchamp et al. 2023a, 2023b; Lebherz et al. 2024b; Vara et al. 2024). Alternatively, there is the possibility of detecting the earliest stage of a gene emergence by studying the emergence of a *de novo* transcribed ORF in individuals or populations. In such a case, the search for homology is conducted against outgroup species, but also against outgroup populations/individuals from the same species, if such data is available (Grandchamp et al. 2023b).

### Homology Filter

One major criterion for identifying a recent *de novo* gene is the lack of homology to any other coding genes outside and inside of the expected phylogenetic group/species/population of emergence. However, we emphasize that the simple absence of homology is not enough to conclusively validate a *de novo* origin. The homology search has to be performed for the full dataset of candidate genes from the previous steps. All of them that show significant homology can then be discarded from the list of potential *de novo* genes.

Each *de novo* gene is required to show no similarity to any gene outside or within the species or taxonomic group of interest, which would suggest that the candidate gene emerged via a recycling mechanism, such as duplication. The inclusion of a greater number of outgroup species in the analysis leads to more robust results.

#### Protein sequences as the default option

The most widely employed method for identifying homologs is to use protein sequence similarity for the purpose of database searches. Such searches may encompass proteins from a broad range of species. Distant outgroup species should be also included to rule out horizontal gene transfer and distant homologies. Large databases containing sequence data from all domains of life, such as the NCBI Reference Sequence Database (Pruitt et al. 2005) can be searched to include as many species and taxonomic groups as possible. Newly assembled genomes and corresponding proteomes that have not been incorporated into public databases can also be beneficial to search when studying a specific taxon (Fig. 2b).

With transcriptome-based analysis, it is often assumed that *de novo* candidates are not annotated in the reference genome. Consequently, annotation software might fail to identify homologous genes in outgroup genomes, leading to incomplete outgroup proteomes. In such cases, validation may rely on the subsequent identification of syntenic homologs that lack coding properties (ex ORFs) or show important frameshift, to confirm the absence of possible homologous encoded protein. Alternatively, Vakirlis and McLysaght (2018) propose performing similarity searches of six-frame translations of entire outgroup genomes. This method discards any putative coding homologs in outgroup genomes, including bona fide noncoding homologs that lacks stop, frameshift and transcription. While this approach is likely to be the most effective, it is more suitable for small genomes, as it can be computationally intensive for larger genomes. The homology search is typically conducted using the protein sequence of the genes to be tested. However, there has been an increasing trend in the use of protein structure, in addition to the sequence, depending on the specific biological question being investigated (Alvarez-Carreño et al. 2021; Middendorf et al. 2024; Van Kempen et al. 2024).

#### Using the DNA sequence to include noncoding RNAs

A homology search can also be performed based on the DNA sequence of candidate *de novo* genes. This can be useful when looking for homology in noncoding RNA (ncRNAs). In such instances, the direction of the alignment should be considered, as well as the coverage, given that two overlapping transcripts could have originated from distinct promotors (Grandchamp et al. 2023a). Furthermore, according to the biological question, it can be wanted that a *de novo* gene is not derived from a transposable element (TE), or from an annotated and conserved ncRNA. To address this, the ORF or transcript can be searched for homology against a database, comprising TEs and ncRNAs from query and outgroup species. An important caveat is that, if proteogenomic evidence of translation exists for a given genomic sequence (Slavoff et al. 2013; Chen et al. 2020; Duffy et al. 2022; Mudge et al. 2022) then such direct evidence overrules the similarity with a long noncoding RNA (lncRNA), and may in fact indicate that the lncRNA is in fact coding (Prensner et al. 2021). Importantly, the use of DNA sequences can be problematic for *de novo* genes that emerged through specific mechanisms such as overprinting or antisense emergence. More precisely, such candidates might exhibit significant DNA similarity with genes they overlap with, leading to their erroneous exclusion from a list of potential *de novo* genes.

#### Available tools for sequence similarity searches

Several tools are available to search for homologous sequences. BLAST (Altschul et al. 1990) is commonly used for homology searches and is recommended because of its speed and accuracy. When working with a large database such as the NCBI nr or RefSeq, a faster tool for local alignments than BLAST, such as Diamond (Buchfink et al. 2021), can be used. As *de novo* genes that show homology to existing proteins should be removed from the dataset of potential *de novo* genes, the choice of homology criteria is important.

For highly divergent proteins, algorithms such as BLAST have been shown to be prone to false negatives (Moyers and Zhang 2016, 2017; James et al. 2021). Other algorithms based on Hidden Markov Models (HMMs) have demonstrated greater sensitivity in detecting distant homologies. HMM-based approaches, such as PSI-BLAST (Altschul and Koonin 1998), HMMER3 (Finn et al. 2011), and JackHMMER (Johnson et al. 2010), offer improved handling of insertions and deletions (Eddy 2011). For example, GenEra (Barrera-Redondo et al. 2023) integrates HMM-based methods alongside BLAST to enhance the detection of distant homologs.

Different E-value thresholds can be used to assess homology (Vakirlis et al. 2020), even though an e-value of 10e-2 should be the highest tolerated. For example, one might want to be extremely restrictive while studying one single *de novo* gene involved in a specific function to ensure that it contains no other gene overlap. A more relaxed threshold can be applied if the phylogenetic group includes a lot of species and the homology search is performed against very distant species. An additional measure is the alignment coverage (Long and Langley 1993; McLysaght and Hurst 2016) (Fig. 2b, Table 1).

#### Predicting protein structures for homology searches

Recent advancements in protein structure prediction, most importantly by AlphaFold2 (Jumper et al. 2021), have led to new opportunities for phylogenetic analyses based on protein structures (Moi et al. 2025). Protein structures exhibit greater conservation compared to their sequences (Illergård et al. 2009), suggesting the potential of putative *de novo* genes actually representing highly divergent orthologs (Casola 2018). To further confirm a *de novo* origin, structural similarity searches can be conducted using tools such as Foldseek (Van Kempen et al. 2024). Foldseek enables rapid comparison of structural similarities across a broad range of databases, encompassing both experimental and computationally derived structures. It is important to note that the identification of *de novo* genes should be based primarily on phylogenomic evidence of their recent emergence from noncoding sequences, rather than on structural uniqueness. Even if a protein product of a confirmed de novo gene shows structural similarity to existing proteins, this does not negate its putative de novo origin. Such structural convergence may reflect similar selective pressures leading to analogous three-dimensional solutions or structures easy to reach within sequence-structure space, despite independent evolutionary origins. However, the commonly used AlphaFold2 (Jumper et al. 2021) primarily relies on co-evolutionary data derived from multiple sequence alignments (MSAs), which are inherently sparse for *de novo* proteins, impacting the reliability of predictions (Fig. 2b, Table 1) (Jumper et al. 2021; Aubel et al. 2023; Liu et al. 2023). Given this limitation, there has been growing interest in structure predictors that utilize protein language models. These models are supposedly more suitable for predicting the structures of *de novo* proteins and other orphan proteins, where sequence homologies are limited or nonexistent (Chowdhury et al. 2022; Michaud et al. 2022; Aubel et al. 2023; Lin et al. 2023; Liu et al. 2023; Middendorf and Eicholt 2024). However, it is important to note that both AlphaFold2 and protein language model-based tools, such as ESMfold, have been shown to inaccurately predict structures of *de novo* proteins, and with discordant confidence scores (Aubel et al. 2023; Middendorf and Eicholt 2024). The most recent implementation of AlphaFold—AlphaFold3 (Abramson et al. 2024)—has yet to be tested for its performance on orphan proteins and *de novo* emerged proteins. Recent studies have successfully utilized molecular dynamics (MD) simulations as refinement to explore the structural dynamics of*de novo* proteins (Lange et al. 2021; Middendorf et al. 2024; Peng and Zhao 2024).

After the homology filtering step, the list of candidate genes is reduced to a list of potential *de novo* genes, containing only genes that don’t have detected homologs outside the studied taxonomic group.

### Noncoding Homologs

The detection of syntenic noncoding sequences, homologous to all potential *de novo* genes under investigation, in target species or populations that are outgroup to the ones expressing the potential *de novo* genes, is for now the last step to provide evidence for a *de novo* emergence. In this review, we define a “noncoding homolog” as a homologous sequence that supports the validation of a *de novo* gene emergence. However, determining whether a genomic sequence is truly noncoding can be challenging. As a result, several studies define noncoding homologs as sequences lacking an open reading frame (ORF) that could encode a protein homologous to the one produced by the *de novo* gene (Vakirlis and McLysaght 2018; Sandmann et al. 2023; Wacholder et al. 2023). In such cases, an insertion in the homologous sequence would not necessarily prevent translation, but result in a different frame and with that loss of protein homology.

However, identification of syntenic regions and a coding status can be challenging, and the absence of a “syntenic noncoding homolog” does not necessarily invalidate a *de novo* origin.

The *de novo* origin of a potential *de novo* gene can be suspected under the following conditions:

- homologous sequences to the *de novo* gene can be detected in genome of several target species or populations. Such target species or populations must be outgroup to the phylogenetic group, species or population where the *de novo* genes under investigation are present.
- the identified homologous sequences are noncoding, or would encode a protein sufficiently different from the one encoded by the candidate, for example due to a frameshift early in the sequence.
- the identified homologous sequences are in a genomic location that is syntenic to the *de novo* gene

The following steps are required to detect syntenic noncoding homologs:

#### Selection of Target Genomes for Synteny Search

In order to identify syntenic noncoding homologs, a set of target genomes must be selected. This set of target genomes will be used to validate or invalidate a *de novo* emergence for all remaining genes from the previously filtered set. For instance, in the case of studying *de novo* genes first steps of emergence within a species, the target genomes should be those from individuals or populations of the same species that do not contain the *de novo* gene(s) of interest. Conversely, when searching for *de novo* genes specific to a taxonomic group that includes several species, the target genomes should be closely related to that taxonomic group, but have diverged earlier than the root of this group. The optimal number of target genomes required for the identification of noncoding homologs remains undetermined; however, it is generally accepted that the greater the number of genomes analyzed, the more robust the conclusions drawn (Fig. 2c  Table 1).

The choice of outgroup genomes is not only crucial but also requires careful interpretation of the results. When the selected outgroups are too distantly related to the query genome, the absence of homologous sequences should not be taken as definitive evidence for bannishing a de novo origin. More critically, when only a few distantly related and poorly annotated genomes are available, the syntenic regions may span large genomic intervals due to a low density of annotated genes. In such cases, the likelihood of identifying false nongenic homologs increases, particularly given the small size of many de novo genes. As demonstrated by Roginski et al. (2024), when synteny searches were conducted using large genomic windows (e.g. ten neighboring genes), nearly 100% of candidate de novo genes appeared to have homologs in outgroup genomes. In contrast, using more narrowly defined syntenic regions reduced this number to below 50%, indicating a high rate of false positives in the broader search context.

#### Homology Search between the Query *de novo* Gene and the Target Genomes

Once the target species have been identified, genomic sequences homologous to the potential *de novo* gene can be searched for. During this step, the homology search is performed against the genome of all target species. One option is to use tBLASTn, by using the *de novo* translated ORF as a query (Vakirlis and McLysaght 2018). However, the most precise option to detect homologous sequences independently of their frame of translation is to use BLASTn. If the ORF is small, and if the unspliced gene contains one or several introns, an option is to use the unspliced ORF as a query for a nucleotide BLAST against the target genome, and then splice the resulting alignment (Grandchamp et al. 2023b). If the target genome belongs to a species that is phylogenetically distant from the query species, alignment programs that allow more divergence such as exonerate (Slater and Birney 2005) can also be used to search for homology.

#### Search for Syntenic Regions

Genomic synteny refers to the conservation of genomic fragments within two genomes or chromosomes. If one or several homologous hits have been detected for a single query *de novo* gene, some of these hits can be further validated in each target species by confirming their location in a genomic region that is syntenic to the *de novo* gene. This step can also be performed in reverse with the previous one, meaning that the search of homologous sequences could also be performed only in syntenic regions.

##### Methods for synteny detection

There are numerous methods available for synteny detection. Synteny can be compared between two complete genomes by fragmenting each chromosome into blocks based on sequence fragments, motifs, domains, etc., and determining similarity and location between blocks (Wang et al. 2012; Liu et al. 2018). Synteny can also be examined at a genic level by studying the conservation of the order of syntenic genes between genomes. In such cases, genes are selected as anchors to determine synteny, and the detection of synteny is based on gene orthology. For instance, SynChro (Drillon et al. 2014) and Synima (Farrer 2017) are software tools that detect synteny using reciprocal BLAST hits between genes from different genomes. Using genes as anchors for synteny is a rapid and effective approach when searching for syntenic hits of *de novo* genes that are intergenic (Vakirlis et al. 2020; Roginski et al. 2024). The genes neighboring the *de novo* gene are chosen as anchors and investigated for orthology in the target genome. If the noncoding homolog is flanked by genes orthologous to those surrounding the query *de novo* gene, the synteny is confirmed. The number of anchor genes can be adjusted based on the context. When working within populations or individuals of a single species or closely related species, a stringent requirement for complete synteny may be imposed. In such cases, noncoding sequences homologous to the candidate *de novo* gene are collected only if they are positioned between two genes homologous to those surrounding the query candidate. Other approaches also exist for synteny detection.Käther et al. (2023) introduced an approach called “Annotation-Free Identification of Potential Synteny Anchors” that does not rely on genes as anchors. Zhao and Schranz (2017) suggested using network approaches to infer synteny. One of the best ways to validate synteny is to use whole-genome alignments. In such cases, the genomic region of target genomes that aligns to the *de novo* candidate from the query genome corresponds to the syntenic homolog. For instance, Wacholder et al. (2023) aligned syntenic conserved blocks to precisely locate the coordinates of noncoding homologs compared to candidate *de novo* genes in yeasts. Similarly, Sandmann et al. (2023) used a whole-genome alignment of 120 mammalian species and another alignment of 27 primate species to search for noncoding sequences homologous to human-translated micropeptides. Whole-genome alignments have also been used to identify *de novo* genes in *Drosophila* (Peng and Zhao 2024), though some appear to have been overlooked (Guay et al. 2025). Overall, whole-genome alignments are highly reliable but require several, high-quality genomes, which are often not available.

##### Caveats when using synteny

While validating synteny between *de novo* candidates and homologous sequences is necessary, this steps also is affected by methodological limitations. The definition and conservation of synteny depends on several criteria, such as the quality of genome annotation, alignments, and the selection of syntenic anchors, windows, and algorithms. Liu et al. (2018) demonstrated that synteny between species can be underestimated by up to 40% depending on the methodology chosen. Moreover, once a syntenic block is detected between a query and a target genome, the identification of a noncoding homolog also depends on the methodology. Therefore, the methodology used to detect and define synteny can vary from one project to another, leading to variable conclusions. Independently of the method used, the phylogenetic distance between the query genomes and selected target species influences synteny conservation: the greater the distance between genomes, the less conserved the synteny (Lemoine et al. 2007). For instance, macrosynteny tends to be preserved for approximately 10–100 million years, whereas microsynteny can remain conserved over several hundred million years. For example, many genes are syntenic within Chordates and Arthropods, each of which emerged around 560 million years ago (mya), but not between the two phyla (Vonica et al. 2020), which diverged approximately 708 mya (Kumar et al. 2022). Furthermore, synteny conservation can vary among taxa (e.g. plants, animals) even for similar phylogenetic distances (Roginski et al. 2024). Moreover the detection of syntenic noncoding sequences homologous to *de novo* genes often fails due to factors such as extensive genomic rearrangements. When validation of *de novo* emergence through the detection of a noncoding homolog cannot be achieved, drawing conclusions about *de novo* emergence becomes challenging. Some genes that emerge after a duplication event have been observed to evolve rapidly, diverging from their original sequence to an extent that no homology tool can reliably predict their origin (Gu et al. 2005; Pegueroles et al. 2013; Naseeb et al. 2017; Casola 2018; O’Toole et al. 2018). Consequently, such genes may exhibit no homology to any other annotated gene and could be mistakenly identified as *de novo* genes, in the absence of noncoding homolog (Weisman et al. 2020).

#### Assess the Coding Status of the Detected Homologous Sequences

Once a syntenic homolog of a potential *de novo* gene has been detected, the final step is to determine its coding status. To do so, the query sequence and its homolog are often re-aligned before deeper investigation (Sandmann et al. 2023; Wacholder et al. 2023; Peng et al. 2024). If one homolog shares the same coding properties as the potential *de novo* gene, then such gene did not emerge *de novo*, or at least not prior to the divergence of the two studied species (query and target). On the other hand, if all homologous sequences are noncoding, then the *de novo* origin of the *de novo* candidate under investigation is assumed as the “most likely” in the query species.

Assessing the coding/noncoding status of detected homologs remains the most challenging step of the entire pipeline. Several properties can be assessed to compare the coding status of the sequence homologous to the potential *de novo* gene, such as the presence of start and stop codons, premature stop codons, frameshift mutations, and splice sites in the case of introns (Grandchamp et al. 2023b). However, the question remains: are these features, or their absence, sufficient to validate or invalidate a coding gene status? For example, the absence of an ATG start codon in a noncoding homolog to a *de novo* candidate does not necessarily prevent translation, as several weaker start codons have been shown to be adequate for translation (Cao and Slavoff 2020), with some being conserved across evolution (Bazykin and Kochetov 2011). More precisely, several small peptides have been shown to be often encoded by sORFs with non-AUG start codons (Peng et al. 2024). Wacholder et al. (2023) emphasize frameshift mutations as crucial features to consider, since the position of a frameshift in a putative noncoding homolog can significantly affect the divergence from the *de novo* candidate if both are translated. In Sandmann et al. (2023), authors translated the homologous ORF, if any, and calculated a score of protein homology.

Evaluating transcription of the noncoding homolog also improves the determination of a genic status. Transcription information is also useful for inferring the emergence of splice sites. Several studies have reported the presence of introns in *de novo* genes (Wu et al. 2011; Zhang et al. 2019; Grandchamp et al. 2022). Studying the emergence of these introns and the evolution/conservation of their splice sites would be essential, as the loss or gain of splicing could significantly alter the translated protein. To the best of our knowledge, such a study has not yet been conducted.

This last step must be conducted with caution, as it can lead to significant misinterpretations. Robust conclusions can only be acquired if several strategic target genomes are selected—the more, the better. The transition from a noncoding sequence to a protein-coding gene follows various steps (McLysaght and Guerzoni 2015; Ruiz-Orera et al. 2017). All mutations and transitions can occur in different orders (Carvunis et al. 2012; Iyengar et al. 2024; Lebherz et al. 2024b). More importantly, the process of acquiring a coding status can go back and forth during evolution, as the initial stages of *de novo* emergence are *a priori* not subject to selection pressures (Carvunis et al. 2012; Iyengar and Bornberg-Bauer 2023). Therefore, the detection of noncoding sequences homologous to a candidate *de novo* gene, can only be valuable if such a noncoding status is confirmed in several target, as a coding homolog could hypothetically also be detected in more divergent species that were not studied (Fig. 2c  Table 1).

After all these steps, among the set of potential *de novo* genes under investigation, the ones that have noncoding syntenic homologs in all target genomes can be validated as *de novo* genes.

#### Evolutionary Information

What selective pressures apply on a *de novo* gene? According to the model proposed in 2012 (Carvunis et al. 2012), the emergence of a new gene from a noncoding sequence involves two main steps: the first is the emergence of a proto-gene, which is a transcribed and translated ORF whose genomic sequence is not yet under selection, producing a small peptide that is likely gained and lost through evolution. The second stage is when a proto-gene becomes fixed in a species due to selection, achieving the status of a *de novo* gene (Van Oss and Carvunis 2019). It is challenging to determine whether a *de novo* gene is fixed in a species, and by that gaining a *de novo* gene status, or whether it is not yet fixed, classifying the gene as a proto-gene. Measurements of selection pressures can be used (Feldmeyer et al. 2024) to distinguish between these two. Moreover, the method used to detect *de novo* genes influences of which type the majority of candidate genes are.

*De novo* genes extracted from an annotated genome are likely to become fixed or are fixed already, as their coding features are robust enough to be detected by standard annotation methods. Several studies have demonstrated that *de novo* genes extracted from annotated genomes are under purifying selection both within and between species (Li et al. 2010; Palmieri et al. 2014). Moreover, specific codons have been shown to be enriched in such *de novo* genes (Hershberg and Petrov 2008; Wallace et al. 2013; Schlötterer 2015).

Assessing *de novo* genes extracted from transcriptomes and/or proteomes is more challenging. Labeling such sequences as *de novo* genes should be supported by evidence of purifying selection, conservation within populations of a species and translational evidence. If no selection tests are performed, the term proto-gene is most commonly used. The term ORFans (Vakirlis and McLysaght 2018) or newly expressed ORFs (Grandchamp et al. 2023b) is used for ORFs that were extracted from transcriptomes without evidence of translation. Newly translated ORFs is the commonly used term for ORFs with evidence of translation whose level of transcription is unknown. However, the validation of a *de novo* status does not have to be supported by all these conditions. For instance, in the case of genes annotated by *ab initio* methods, evidence of transcription is generally not provided, unless additional laboratory experiments are conducted. Moreover, *ab initio* and homology-based methods do not provide evidence of selection for the identified genes (Burge and Karlin 1997; Kryazhimskiy and Plotkin 2008). Conversely, if an unannotated ORF exhibits direct evidence of both transcription and translation, there is no conceptually valid reason to apply more restrictive criteria than for canonical genes.

Unfortunately, assessing evidence of selection in *de novo* genes remains extremely challenging (Fig. 2d  Table 1). Selection pressure is often assessed using metrics such as the *dN/dS* ratio (Yang and Bielawski 2000; Hurst 2002; Kosakovsky Pond and Frost 2005) or the *pN/pS* ratio (McDonald and Kreitman 1991). However, both of these metrics are designed for coding sequences. Therefore, the presence of noncoding homologs or noncoding variants of a *de novo* emerged ORF poses problems for their calculation. While these difficulties do not prevent the study of selection among all coding samples of a *de novo* emerged ORF, a future challenge would be to incorporate noncoding sequences into a calculation of selective pressure, to gain a clearer understanding of selection dynamics in the earliest stages of emergence.

Lastly, most *de novo* ORFs are shorter than canonical ORFs and are present in a limited number of species or populations, which limits the statistical power to confidently detect selection (Wacholder et al. 2023). Several studies have addressed the challenge of assessing selection on *de novo* emerged ORFs. For example, Ward and Kellis (2012) attempted to understand whether the large portion of the human genome that is biochemically active shows evidence of purifying selection. By using genome alignments and studying sequence conservation, they found that 4% of the human genome is subject to lineage-specific constraint, in addition to the 5% already known. In 2003, Kellis et al. (2003) developed a reading frame conservation (RFC) test to classify all ORFs of *S. cerevisiae* as either biologically meaningful or meaningless. This RFC test was later adapted by Wacholder et al. (2023) to distinguish ORFs evolving under selection from other ORFs in the yeast genome particularly those showing weak signals in more classical selection tests. While they found no evidence of purifying selection acting on most of these *de novo* emerged ORFs, a few samples showed selection.

### Available Software

The identification of *de novo* genes is contingent on numerous methodological decisions, with custom scripts or programs frequently required for multiple steps in the process. Fortunately, recent advancements have led to the publication of various tools and software that automate *de novo* gene detection, either completely or partially. Singh and Wurtele (2021) developed *orfipy*, which facilitates the detection of ORFs in new transcriptomes that can be used subsequently to search for *de novo* genes in transcriptomic data. The R package *phylostratr* (Arendsee et al. 2019b) allows to infer a phylostratum for all input query genes, thereby enabling the identification of homology to a candidate gene. GenEra (Barrera-Redondo et al. 2023) allows to detect taxonomically restricted genes. The softwares *fagin* partially automate (Arendsee et al. 2019a) and DENSE (Roginski et al. 2024) automate the detection of *de novo* genes in an annotated genome. An automated tool for detection of *de novo* genes based on transcriptomic data is unfortunately not yet available.

### Challenges & Conclusions

In conclusion, despite significant advances in understanding *de novo* gene emergence, two major challenges remain. Firstly, current methods for detecting *de novo* genes are largely limited to evolutionary young genes, making it difficult to discern the origins of ancient genes within large and complex gene families. This limitation stems from the fact that existing approaches can only trace the recent origin of a gene, which becomes increasingly challenging as the gene ages and undergoes multiple rounds of duplication and divergence of sequence and function. As a result, our current understanding of *de novo* gene emergence is biased towards recently evolved genes, leaving a significant gap in our knowledge of how older *de novo* genes originated. Novel approaches for remote homology detection and improved structure predictions could help us address this bias in the future.

Secondly, the lack of standardization in methodology and terminology hinders comparability between studies, with different approaches and thresholds yielding disparate results even when analyzing the same species. We address this problem directly in our accompanying paper by providing a standardized annotation format based on the identified classifications described in this review. Such a standardized annotation format represents a crucial step towards achieving a common framework, enabling researchers to compare and build upon each other’s work more effectively.

By establishing a common framework for describing, analyzing and comparing *de novo* gene studies, we can enhance reproducibility, comparability, and ultimately, drive progress in this rapidly evolving field. Albeit the remaining challenges in this young field, our work paves the way for future studies to refine methods and integrate *de novo* gene searches into standard gene annotation pipelines, unlocking new biological insights into the origins of genes.

## Data Availability

All data and source code for the developed annotation format based on this work can be found at https://github.com/EDohmen/denofo.
